# Simulation im Wahlfach Rechtsmedizin im Dritten Abschnitt der Ärztlichen Prüfung

**DOI:** 10.1007/s00194-022-00586-w

**Published:** 2022-07-19

**Authors:** Dietrich Stoevesandt, Lina Woydt, Jonas Steglich, Rüdiger Lessig, Mathias Rudzki, Axel Schlitt, Marko Weber

**Affiliations:** 1 Dorothea Erxleben Lernzentrum, Medizinische Fakultät, Martin-Luther-Universität Halle-Wittenberg, Magdeburger Straße 12, 06112 Halle (Saale), Deutschland; 2 Institut für Rechtsmedizin, Medizinische Fakultät, Martin-Luther-Universität Halle-Wittenberg, Halle (Saale), Deutschland; 3 Zentrale Notaufnahme, Universitätsklinikum Halle (Saale), Halle (Saale), Deutschland; 4 Abteilung für Kardiologie und Diabetologie, Paracelsus-Harz-Klinik Bad Suderode, Quedlinburg, Deutschland; 5 Medizinische Fakultät, Martin-Luther-Universität Halle-Wittenberg, Halle (Saale), Deutschland

**Keywords:** Staatsexamen, Todesbescheinigung, Leichenschau, Todesursache, Simulator, State examination, Death certificate, Postmortem examination, Cause of death, Simulator

## Abstract

In der COVID-19-Pandemie konnte der Dritte Abschnitt der Ärztlichen Prüfung an Simulationspersonen und Simulatoren durchgeführt werden. Auch im Fach Rechtsmedizin ist deren Einsatz sinnvoll, da eine höhere Standardisierung und Vergleichbarkeit von Prüfungsleistungen erreicht wird und die Verwendung von echten Leichnamen aus medikolegalen Gründen oft nicht vertretbar ist. Der vorliegende Fall berichtet über die Vor- und Nachteile einer Simulation im Staatsexamen, in der anhand einer Leichenschau am Simulator und einer Fremdanamnese eine Todesbescheinigung vollständig ausgefüllt werden sollte.

## Abstract (en)

During the COVID-19 pandemic the third section of the medical examination could be performed on simulation patients and simulators. Their use is also beneficial in forensic medicine, as a higher level of standardization and comparability of examination performance is achieved, and the use of real corpses is often not justifiable for medicolegal reasons. This case reports on the advantages and disadvantages of a simulation in the state examination in which a death certificate was to be completely filled out on the basis of an external postmortem examination on the simulator and an external anamnesis.

## Hintergrund

Gemäß der Approbationsordnung für Ärzte (ÄApprO) [2] wird das Medizinstudium mit dem Dritten Abschnitt der Ärztlichen Prüfung (M3) abgeschlossen. Durch die „Verordnung zur Abweichung von der Approbationsordnung für Ärzte bei einer epidemischen Lage von nationaler Tragweite“ [3] wurde im Rahmen der COVID-19-Pandemie die mündlich-praktische Prüfung an Simulationspatienten (SP) und Simulatoren auch in Deutschland ermöglicht, wie es in anderen Ländern bereits üblich ist [5].

Simulationspersonen sind schauspielerisch begabte Menschen, die nach einer Schulung die Rolle eines Patienten in variablen, standardisierten Situationen einnehmen, ohne dass eine misslungene Interaktion echte Patienten belastet.

Insbesondere im Fach Rechtsmedizin ist die Verwendung von echten Patienten oder Leichnamen im Kontext von Gewalt- und Rohheitsdelikten aus ethischen, forensischen oder organisatorischen Gründen oft nicht vertretbar.

Es gibt bereits einige Ansätze, rechtsmedizinische Lehre und Prüfungen durch die Verwendung von Simulatoren und SP zu standardisieren und ohne die Arbeit am realen Leichnam authentisch zu prüfen bzw. zu lehren [6]. Durch die vorübergehende Änderung der ÄApprO konnten diese nun erstmalig auf die M3-Prüfung übertragen werden.

Alle Medizinstudierenden der Martin-Luther-Universität Halle-Wittenberg wurden 2020 und 2021 mittels Einzelprüfung in einem Fall aus Chirurgie, innerer Medizin oder Wahlfach geprüft. Der Prüfungsstandard umfasste ein 30-minütiges Anamnesegespräch, eine darauf aufbauende diagnostische Strategie und nach Erhalt einer Patientenakte die Erstellung einer Epikrise mit Therapieplan. Die getätigten Anforderungen und die Epikrise wurden den Prüfern vor Prüfungsbeginn ausgehändigt. Die Prüfer konnten die Simulation per Videoübertragung verfolgen. Im Anschluss an die Simulation erfolgte das eigentliche bis zu 60-minütige Prüfungsgespräch vor der dreiköpfigen Prüfungskommission.

Anhand eines rechtsmedizinischen Prüfungsfalls sollen die Vor- und Nachteile einer simulationsgestützten M3-Prüfung dargestellt und kritisch diskutiert werden.

## Falldarstellung

### Simulationsszenario

Der Prüfling befand sich zu Simulationsbeginn mit einer SP im kameraüberwachten Simulationsraum. Die SP spielte eine Pflegeperson, welche angab, den Patienten aus den vorherigen Diensten zu kennen und am Morgen tot im Bett aufgefunden zu haben, so konnte eine Fremdanamnese des Verstorbenen erhoben werden.

Informationen zu vorangegangen Untersuchungen konnten der Patientenakte entnommen werden. In der schriftlich gestellten Prüfungsaufgabe sollte eine Todeszeitschätzung durchgeführt und zusammen mit der Epikrise eine Todesbescheinigung im Original ausgefüllt werden. Dies entspricht den Lernzielen V.01.1.1.148 und VIII.7-02.6.4. des NKLM [7]. Hierfür standen nach der Simulation weitere 45 min zur Verfügung.

**Figure 1:**
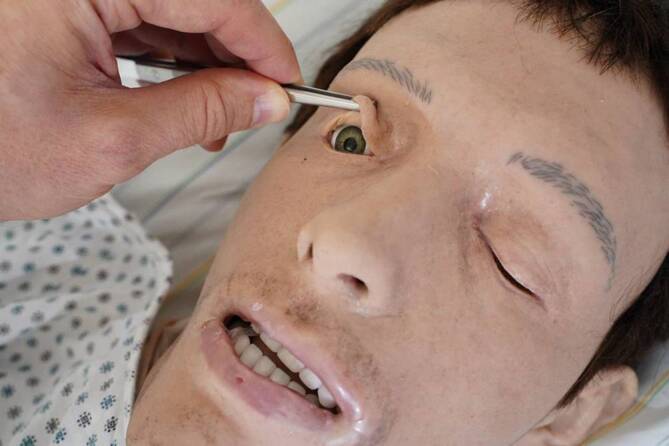

**Figure 2:**
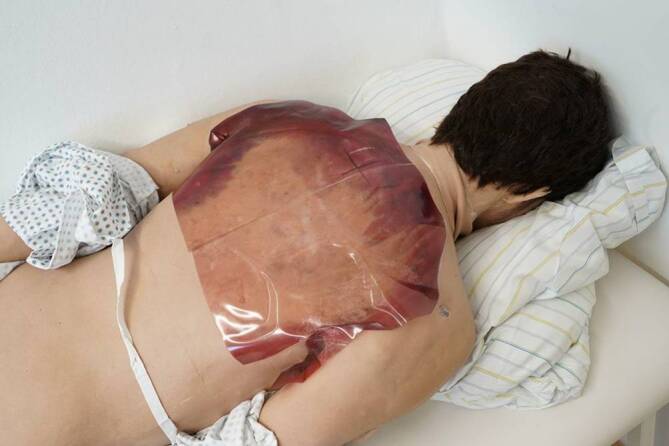

Als Leichenmodell wurde ein Reanimationssimulator der Fa. Skillqube (Wiesloch, Deutschland; Abb. 1), männlich, mittelalt, mit materialbedingter vorhandener Totenstarre verwendet. Die Totenflecken (Abb. 2) wurden auf dem Rücken aufgeklebt und der Prüfling schriftlich über die unvollständige Wegdrückbarkeit informiert. Der Simulator lag mit einem dünnen Pflegehemd bekleidet unter einer leichten Decke im Patientenbett.

### Angaben zum Patienten

Der am Vortag zur Lymphknotenexstirpation stationär aufgenommene 55-jährige Patient (72 kg, 175 cm) mit bekanntem malignem Melanom wurde am Morgen um 7.30 Uhr tot im Bett aufgefunden, nachdem er zuletzt um 22.00 Uhr lebend gesehen wurde.

Der Patientenakte konnten folgende Angaben entnommen werden: ein diätetisch geführter Diabetes mellitus Typ 2, eine arterielle Hypertonie und ein paroxysmales Vorhofflimmern (VHF) – entsprechend einem vom Prüfling zu berechnenden CHA_2_DS_2_-VASc-Score von 2 – mit Z. n. Pulmonalvenenisolation vor ca. 8 Wochen, seitdem kein bekanntes Rezidiv des VHF. Außerdem fand sich hier ein EKG vom Vortag mit erneut aufgetretenem VHF und ein im Staging-CT beschriebener Vorhofthrombus im linken Herzohr, jedoch keine dokumentierte Reaktion auf diese beiden Befunde. Bei Aufnahme erfolgte präoperativ die Anpassung der Antikoagulation mit Rivaroxaban, 20 mg, durch Enoxaparin, 40 mg, einer bei VHF und Vorhofthrombus aus kardiologischer Sicht inadäquaten Antikoagulation. Zusätzlich wurden Bisoprolol 2,5 mg (1-0-0) und Ramipril 5 mg (1-0-0) gegeben. Im Staging-CT/-MRT fanden sich keine Metastasen. Das Aufnahmelabor zeigte außer gering erhöhten D‑Dimeren einen Normalbefund.

Die Körpertemperatur tief rektal betrug 33 °C und wurde von der anwesenden SP mitgeteilt, da der verwendete Simulator kein Rektum aufwies. Die Raumtemperatur von 20 °C konnte über ein vorhandenes Thermometer abgelesen werden. Damit sollte ein geschätzter Todeszeitpunkt vor 5,2–10,8 h vom Prüfling mittels Lineal und Henssge-Nomogramm unter Berücksichtigung eines Korrekturfaktors (KF) von 1,1 bei unbewegter Luft und einer bis 2 dünnen Bekleidungsschichten bestimmt werden; ein KF von 1,2 wurde auch als richtig gewertet.

Unter 2 Pflastern fanden sich Hautnähte der Lymphknotenexstirpation in der linken Axilla bzw. der Entfernung des Primärtumors am linken Oberarm, die Inspektion zeigte keine weiteren pathologischen Befunde.

### Intention des Falls

Der Prüfling sollte die ungeklärte Todesart erkennen oder eine nichtnatürliche Todesart diskutieren sowie die Todesbescheinigung korrekt ausfüllen. Außerdem sollte er ein Todeszeitintervall mit dem Henssge-Nomogramm abschätzen, die wahrscheinlichste Kausalkette (Vorhofthrombus, Änderung der Antikoagulation, Schlaganfall) beschreiben sowie den Anruf bei der Polizei als weiteres Vorgehen benennen können. Den Prüfern wurde freigestellt, auch über eine mögliche ärztliche Sorgfaltspflichtverletzung im Rahmen des Prüfungsgespräches zu diskutieren. Dabei wurde nicht vorausgesetzt, dass der Prüfling die inadäquate Antikoagulation als solche erkannte. Die Durchführung der Leichenschau wurde nicht bewertet.

## Diskussion

Die vorgestellte Form einer simulationsbasierten M3-Prüfung ermöglicht die Prüfung rechtsmedizinischer Lernziele standardisiert und realitätsnah mit vertretbarem Aufwand. Die Durchführung erfolgte analog zu Fallszenarien aus klinischen Fachgebieten entsprechend dem Prüfungsstandard und wurde sowohl von den Prüfern als auch den Prüflingen als fair und interindividuell vergleichbar wahrgenommen [4]. Die beschriebene Simulation bietet die Möglichkeit, die oben genannten Lernziele ohne Rücksicht auf evtl. medikolegale Aspekte eines realen Falles zu prüfen.

Limitationen der gewählten Methode ähneln den von Richter et al. beschriebenen Einschränkungen der auf virtueller Realität basierenden Leichenschau. Zwar lässt sich die Totenstarre am Leichenmodell besser simulieren, jedoch ist man materialbedingt beim genutzten Simulator auf deren Vorhandensein festgelegt. Weitere taktile und olfaktorische Wahrnehmungen lassen sich nicht simulieren [8]. Im verwendeten Leichenmodell kann außerdem eine tiefe rektale Temperaturmessung als potenzielle Fehlerquelle nicht durchgeführt werden. Speziellere rechtsmedizinische Untersuchungstechniken zur Todeszeitschätzung wie Testung des idiomuskulären Wulstes oder der elektrischen Erregbarkeit der mimischen Muskulatur sind derzeit nicht möglich.

In anderen M3-Simulationen konnten weitere rechtsmedizinische Lehrinhalte wie ein Rohheitsdelikt mit Bulbustrauma, ein nichtakzidentelles Trauma und ein Amphetaminabusus geprüft werden.

### Ausblick

Die weitere Erprobung rechtsmedizinischer Fallszenarien erscheint sinnvoll, da die geplante Änderung der ÄApprO den Einsatz von Simulationen und „objective structured clinical examination“ (OSCE) in Staatsexamina in Deutschland dauerhaft ermöglichen könnte. Damit wären realitätsnahe, reproduzierbare und vergleichbare Szenarien unter bestmöglicher Umgehung der an einigen Standorten hürdenhaften Umsetzbarkeit einer Prüfung am realen Leichnam möglich. Denkbar wäre die Prüfung des Ablaufes der Leichenschau als OSCE-Komponente [6], um die Objektivität weiter zu erhöhen. Die Todesbescheinigung könnte computer- [1] oder Paper-Pencil-basiert erfolgen, um den Personalaufwand zu reduzieren.

## Fazit für die Praxis

Da der Einsatz von echten rechtsmedizinischen Fällen im Staatsexamen aus logistischen und/oder rechtlichen Gründen in der Regel nicht möglich ist, stellt die Simulation eine realitätsnahe, gut standardisierbare Prüfungsform für rechtsmedizinische Inhalte dar.
